# A Non-surgical Multimodal Approach to Adolescent Idiopathic Scoliosis (Lenke 5C) Using an Intensive Two-Week CLEAR Institute Protocol: A Report of Two Cases

**DOI:** 10.7759/cureus.102006

**Published:** 2026-01-21

**Authors:** John Whelan, Paris Paige, Justin M Dick

**Affiliations:** 1 Physical Medicine and Rehabilitation, Clear Life Scoliosis Reduction and Chiropractic, Huntersville, USA; 2 Chiropractic, Life University, Atlanta, USA

**Keywords:** adolescent idiopathic scoliosis, case series, clear institute, high-frequency therapy, ligamentous creep, multimodal rehabilitation, neuromuscular re-education, scolibrace

## Abstract

Adolescent idiopathic scoliosis (AIS) is a progressive three-dimensional spinal deformity often managed with physiotherapeutic scoliosis-specific exercises (PSSE), bracing, or spinal fusion. Emerging evidence suggests that multimodal, high-frequency non-surgical rehabilitation may produce measurable improvements in spinal alignment through combined biomechanical loading and neuromuscular re-education.

This case series evaluates radiographic and functional outcomes in two AIS subjects (Lenke 5C) undergoing a two-week intensive CLEAR protocol followed by ScoliBrace® implementation. Two female adolescents, with a mean age of 12 years, presented with rapid curve progression and no prior exposure to bracing or scoliosis-specific therapy. Both completed a two-week, 20-session intensive CLEAR program incorporating mechanical traction, Mirror Image® positioning, proprioceptive neuromuscular training, whole-body vibration, and corrective exercise. ScoliBrace® fitting occurred during week two. Radiographs were obtained at baseline, immediately post-intensive care, and at 6- and 12-month follow-up. Functional outcomes included the Functional Rating Index (FRI), height, chest expansion, angle of trunk rotation, Stork balance testing, and Modified Cox measurements.

Case 1 demonstrated a lumbar Cobb angle reduction from 35.7° pre-intensive to 21.7° post-intensive and 10.4° at 12 months (71% total reduction). Case 2 demonstrated a reduction from 38.9° to 24.7° post-intensive and 15.7° at 12 months (60% total reduction). Both exhibited corresponding improvements in functional metrics.

A short-duration, high-frequency multimodal rehabilitation program combined with custom bracing may produce rapid and sustained radiographic improvements in AIS. These outcomes warrant further investigation through larger prospective studies.

## Introduction

Adolescent idiopathic scoliosis (AIS) is a 3D spinal deformity defined by a lateral curvature of the spine exceeding 10° on standing radiographs, accompanied by vertebral rotation and trunk asymmetry [1]. AIS typically presents between 10 and 17 years of age and affects approximately 2-3% of adolescents, with a marked predominance in females [2,3,4]. Curve progression most commonly coincides with periods of rapid pubertal growth, reflecting the dynamic interplay between spinal loading, ligamentous tension, and neuromuscular adaptation [5]. Left untreated, AIS can lead to pain, progressive deformity, pulmonary compromise in large thoracic curves, and psychological distress related to body image and disability [6,7].

Traditional management strategies for AIS depend on curve magnitude and skeletal maturity. Physiotherapeutic scoliosis-specific exercises (PSSE) are recommended for mild curves under 25°, while bracing is typically indicated for moderate curves between 25° and 60° [8]. Surgical correction, most commonly spinal fusion, is reserved for curves exceeding 45° that continue to progress despite conservative care [9]. However, surgical interventions carry inherent risks, including infection, instrumentation failure, persistent pain, and reduced spinal mobility [9-10]. Additionally, many subjects with surgical-range curves decline operative intervention, seeking non-surgical alternatives that preserve spinal function and minimize procedural risk.

Recent literature supports the efficacy of multimodal non-surgical approaches that combine 3D corrective bracing with targeted neuromuscular and biomechanical rehabilitation [10-11]. Nalda A et al. demonstrated that a structured, non-surgical regimen incorporating ScoliBrace®, scoliosis-specific rehabilitation, and CLEAR Institute corrective methods can significantly reduce Cobb angle and improve trunk symmetry, even in surgical-range curves [10]. Similarly, randomized controlled trials of Schroth-based therapy, such as Zapata KA et al. (2023), have shown radiographic and quality-of-life improvements in adolescents with mild AIS [11]. These findings underscore the emerging role of non-invasive interventions capable of restoring more physiological spinal biomechanics through neuromuscular re-education and controlled mechanical loading.

Building on this foundation, the present case series investigates the outcomes of two AIS cases (Lenke 5C classification) treated simultaneously using a two-week intensive, non-surgical scoliosis reduction program based on the CLEAR Institute protocol that integrates ScoliBrace®. ScoliBrace® is a customized, rigid thoraco-lumbo-sacral brace created using 3D scanning technology to over-correct the spine in three dimensions. It uses a Mirror Image® approach and spinal coupling principles to position the body opposite to the scoliotic deformity, guiding the spine toward maximum possible straightening. The creation process and approach differentiate this brace from other scoliosis bracing options [10].

The CLEAR™ Institute protocol is a combination of therapies and home care that, when combined, aim to elicit motion in restricted areas of the spine, align spinal segments, and retrain feedback loops associated with proprioception, balance, and posture. Inclusion criteria included Lenke 5C classification and consecutive treatment. Exclusion criteria included subjects without a diagnosis of AIS with Lenke 5C classification. The Lenke 5C classification describes a specific pattern of AIS. The designation “5” indicates a single structural thoracolumbar or lumbar curve with two minor, nonstructural compensatory curves, while the modifier “C” denotes that the center sacral vertical line lies completely medial to the apical vertebral body [12].

Following the two-week intensive period, evaluations were performed at 90 days, 6 months, and 12 months for each subject. We hypothesize that the high-frequency intervention during the two-week intensive period leverages the biomechanical principles of ligamentous creep, the time-dependent deformation of viscoelastic spinal tissues under sustained or repetitive load, and neuromuscular re-education, promoting rapid and sustained postural correction [13]

The aim of this report is to describe radiographic and functional changes in two adolescents with Lenke 5C AIS following a two-week intensive CLEAR protocol combined with ScoliBrace® fitting. This investigation evaluates whether short-term, repetitive daily corrective loading and exercise can induce measurable improvements in sagittal and coronal spinal alignment, as well as functional outcomes, over a one-year follow-up period.

## Case presentation

Case 1

A 13-year-old female, Risser 2, diagnosed with AIS, was referred to a chiropractic clinic in Charlotte, NC, from an outside chiropractic office. Upon presentation on June 3, 2024, she reported recent, rapid progression of her spinal curvature and had not previously undergone bracing or scoliosis-specific physical therapy. An initial diagnostic measurement of a 21.9° Cobb angle had been recorded 6 months prior to the intensive program by a previous physician.

Physical examination revealed notable asymmetries of the hips, shoulders, and paraspinal musculature. Orthopedic testing was unremarkable, including negative superficial abdominal reflexes, shoulder depression, foraminal compression, cervical distraction, and straight leg raise tests. Additional objective measures included height, Stork balance test performance, angle of trunk rotation readings in prone and flexion positions, Modified Cox test findings, and chest expansion values. Subjective symptoms were assessed using the Functional Rating Index (FRI) (Table 1).

**Table 1:** Objective clinical and functional measurements at baseline, post-intensive, and 12-month follow-up: Functional Rating Index, height (cm), chest expansion (in), angle of trunk rotation (°), Stork balance test (seconds to failure), and Modified Cox test (degrees). The Functional Rating Index (FRI) integrates elements of the Oswestry Low Back Disability Questionnaire and the Neck Disability Index into a single instrument designed to reduce administrative burden. The FRI is scored on a 0-40 scale, with 0 indicating no functional burden and 40 representing the highest level of disability. Prior studies have validated the FRI, demonstrating strong reliability, validity, and responsiveness, while offering greater efficiency in both clinical and research settings [14].

Measure	Case 1 (June 3, 2024) Baseline	Case 1 (June 14, 2024) Post-intensive	Case 1 (June 14, 2025) 12-month follow-up	Case 2 (June 3, 2024) Baseline	Case 2 (June 14, 2024) Post-intensive	Case 2 (June 2, 2025) 12-month follow-up
Functional Rating Index	9/40	8/40	6/40	16/40	1/40	2/40
Height (in)	55.9	56.5	56.7	63.6	65	65.4
Chest expansion (in)	1.25	1.75	1.5	1.5	2.5	3
Angle of trunk rotation, dorsal (flexion)	0°	0°	1° Right	11° Left	4° Right	0°
Angle of trunk rotation, dorsal, lumbar (flexion)	10° Right	6° Right	5° Right	12° Right	2° Right	1° Right
Angle of trunk rotation, lumbar (flexion)	22° Right	16° Right	4° Left	5° Right	7° Right	9° Right
Angle of trunk rotation, dorsal (prone)	0°	0°	3° Right	3° Left	0°	0°
Angle of trunk rotation, dorsal, lumbar (prone)	10° Right	6° Right	6° Right	3° Left	12° Left	0°
Angle of trunk rotation, lumbar (prone)	22° Right	15° Right	3° Right	15° Right	12° Right	5° Right
Balance test	Bilaterally within normal limits	Bilaterally within normal limits	Bilaterally within normal limits	Bilaterally within normal limits	Bilaterally within normal limits	Bilaterally within normal limits
Stork test (seconds to failure)	Left: 3; Right: 3	Left: 4; Right: 12	Left: 22; Right: 5	Left: 22; Right: 5	Left: 8; Right: 5	Left: 18; Right: 8
Modified Cox test (degrees)	Left: 50; Right: 45	Left: 75; Right: 35	Left: 60; Right: 40	Left: 45; Right: 70	Left: 50; Right: 70	Left: 45; Right: 45

Radiographic evaluation consisted of weight-bearing, full-spine anteroposterior and lateral views assessing both coronal and sagittal alignment. Baseline Cobb angle measurement demonstrated a 35.7° lumbar curve on initial presentation for the intensive program. This indicates a high risk of progression due to skeletal immaturity.

Intervention

Following the initial evaluation, Case 1 completed a two-week intensive program consisting of 20 in-office sessions (2 sessions per day, spanning 2 consecutive weeks, Monday-Friday), following the CLEAR™ Institute’s established “Mix-Fix-Set” model of care. Each session lasted approximately 120 minutes and was supervised by a licensed chiropractor trained in scoliosis biomechanics through the CLEAR™ Institute.

Follow-Up and Evaluation

Post-treatment assessments were performed every 90 days following completion of the intensive program. The 90-day, 6-month, and 12-month evaluations included repeat scoliosis radiographs, Cobb angle measurement, and FRI scoring. Each radiograph was acquired after a minimum 24-hour out-of-brace period. All radiographs were obtained and interpreted by the same physician to maintain consistency in image acquisition and analysis. Case 1 demonstrated measurable improvement in coronal alignment, consistent with previously published findings supporting the CLEAR™ Institute’s multimodal approach to non-surgical scoliosis rehabilitation [15]. Case 1 reported 60% compliance with brace wear and home care.

Results

**Figure 1:**
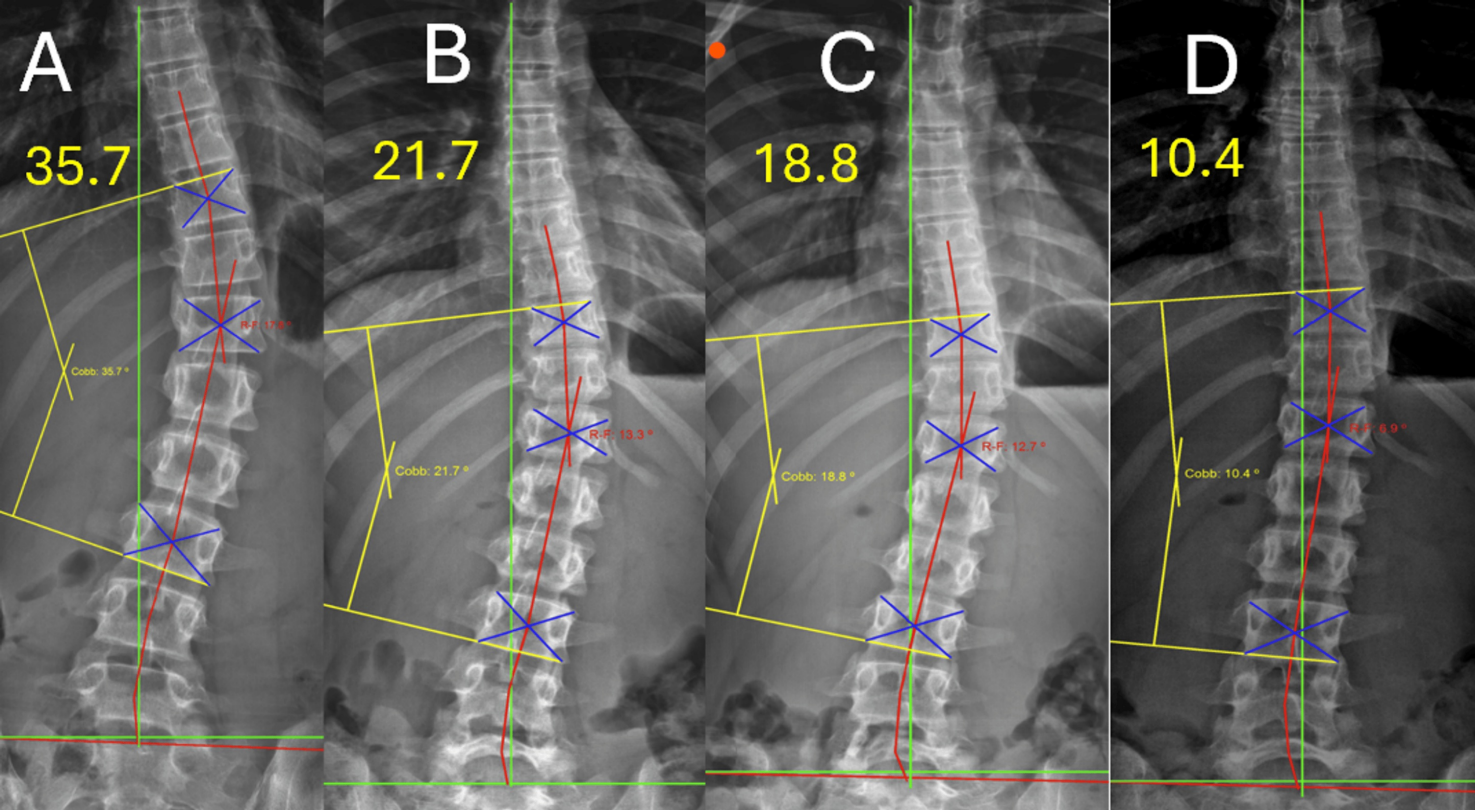
Anterio-posterior (AP) radiographs were obtained at four intervals: pre-treatment (Panel A), prior to intensive care visit 18 on day 10 (Panel B), 6-month follow-up (Panel C), and 12-month follow-up evaluation (Panel D). Yellow lines denote Cobb angle measurements, green lines represent the ideal coronal alignment, and red lines illustrate the Risser-Ferguson analysis (Risser 2). These images demonstrate progressive improvement in Cobb angle magnitude and coronal balance over time. All radiographic measurements were performed using PostureRay® software. A PostureRay® study confirms a high degree of inter- and intra-examiner reliability of line-drawing methods, establishing validity. It was also found that there was no statistical difference in average concordance between hand-drawn and PostureRay® measurements in any region of the spine. All radiographs were digitally measured using PostureRay® software, which uses machine-learning-assisted radiographic parameter mensuration [16]. All measurements and readings of the radiographs were performed by the same physician.

Figure 1A depicts the initial radiographs, with a 35.7° Cobb angle. Figure 1B illustrates the results after 18 treatment visits, with a 21.7° Cobb angle and a 39% reduction (these images were taken prior to any treatment provided on the last day of intensive care, with 2 treatments, or 1 full day, remaining). Figure 1C represents the 6-month follow-up after the 2-week intensive program. Figure 1D illustrates the radiographs at the one-year post-treatment evaluation, with a 10.4° Cobb angle, representing a total reduction of 71%.

Case 2

A 12-year-old female, Risser 2, with a history of prior chiropractic care, presented on June 3, 2024, with documented AIS and recent, rapid progression of spinal curvature. Similar to Case 1, she had no history of bracing or scoliosis-specific rehabilitation before evaluation. A recent measurement of a 30° Cobb angle had been recorded 2 months prior to the intensive program by a previous physician.

Physical examination revealed notable asymmetries of the hips, shoulders, and paraspinal musculature. Orthopedic testing was unremarkable, including negative superficial abdominal reflexes, shoulder depression, foraminal compression, cervical distraction, and straight leg raise tests. Additional objective measures included height, Stork balance test performance, angle of trunk rotation readings in prone and flexion positions, Modified Cox test findings, and chest expansion. Subjective symptoms were assessed using the FRI (Table 1).

Baseline anteroposterior radiographs demonstrated a 38.9° lumbar curve upon initial presentation for the intensive program. This classified the subject as high risk for progression, as she was not skeletally mature.

Intervention

**Figure 2:**
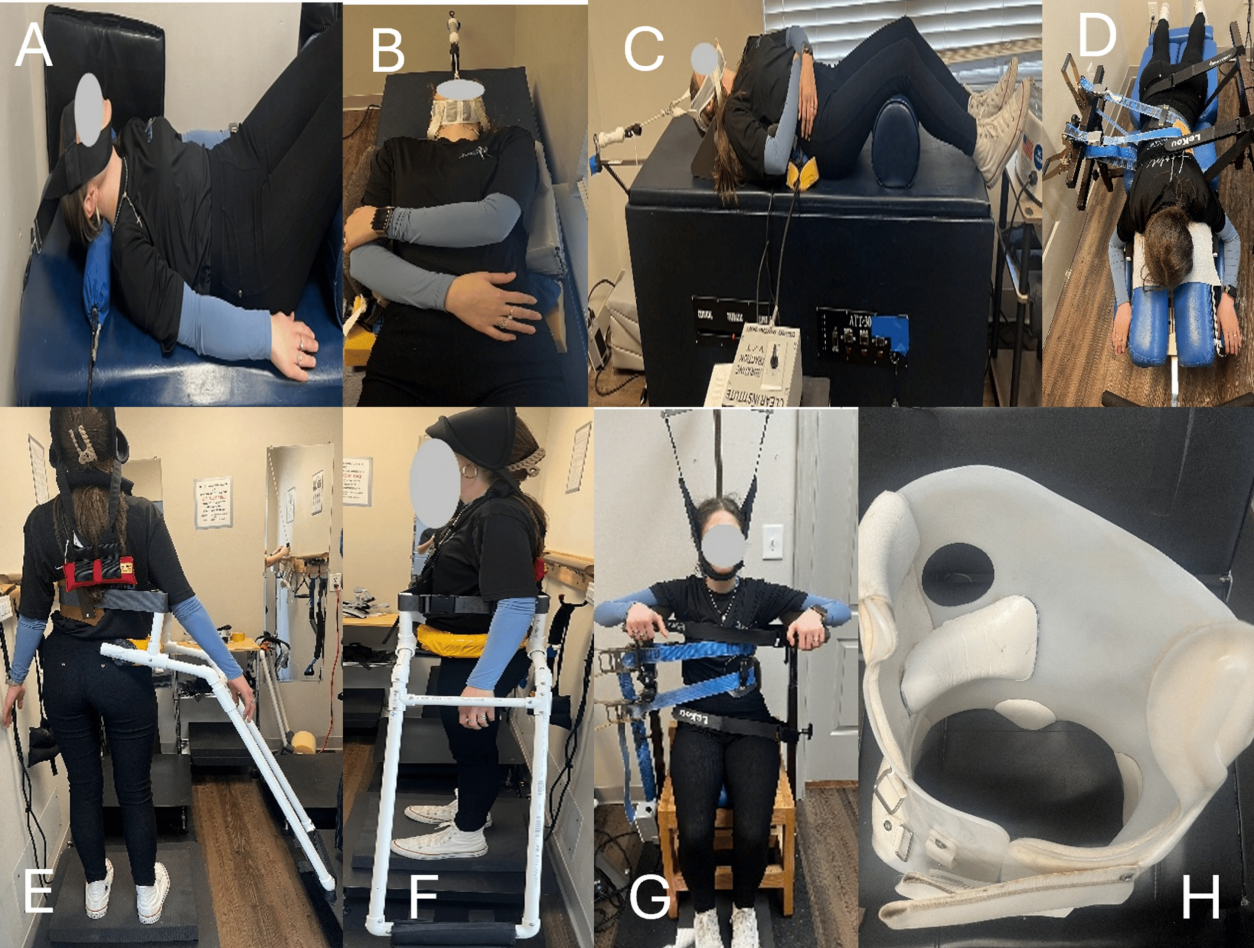
A: Cervical vibrating traction (mechanical traction); B-C: Mechanical drop piece (mechanical traction); D: Flexion/distraction table; E-F: Spinal weighting (proprioceptive neuromuscular re-education); G: Scoliosis traction chair; H: ScoliBrace® 3-D corrective TLSO brace. A: Cervical lordosis was addressed using mechanical vibrating traction, which applies controlled Y-axis distraction to tension cervical soft tissues, including discs and ligaments, and to increase joint spacing. Traction was delivered at 5 pounds with superimposed vibration at 4.5 Hz to facilitate viscoelastic relaxation and allow the tissues to mold to the corrective posture. The intervention was performed for 20 minutes per session. B-C: A controlled joint-mobilization method designed to restore motion to a restricted or stiff articulating segment, with 10 pounds of Y-axis traction. Y-axis mechanical traction involves axial spinal distraction applied along the longitudinal axis to reduce compressive loading and improve spinal compliance. Within protocols taught by the CLEAR Institute, it is used as an adjunctive, preparatory intervention to decrease mechanical resistance and enhance responsiveness to subsequent scoliosis-specific corrective procedures. D: A scoliosis-specific therapeutic table designed to apply low-force, intermittent traction to facilitate gentle elongation and mobilization of the scoliotic spine. E-F: This approach engages intrinsic postural control pathways by using external weighting to elicit compensatory righting responses. Repeated exposure reinforces these corrective patterns, facilitating long-term neuromuscular and postural re-education. G: A seated traction method designed to deliver low-force spinal unloading, during which the spine is progressively de-rotated, elongated, and realigned to counter scoliotic deformity. H: ScoliBrace® is a customized rigid TLSO designed to apply 3D, overcorrective forces, positioning the patient in an in-brace alignment intended to counteract spinal deformity and postural imbalance using a Mirror Image®-based approach. TLSO: Thoracolumbosacral orthosis.

Case 2 received the same CLEAR™ Institute multimodal, non-surgical “Mix-Fix-Set” protocol (Figure 2). The full rationale and procedural details of the CLEAR™ method are outlined after the Results section of this case in the manuscript and in prior institutional publications.

Follow-Up and Outcomes

Post-treatment assessments were performed every 90 days following completion of the intensive program. The 90-day, 6-month, and 12-month evaluations included repeat scoliosis radiographs, Cobb angle measurement, and FRI scoring. Each radiograph was acquired after a minimum 24-hour out-of-brace period before images were taken. Case 2 demonstrated measurable improvement in coronal alignment, consistent with previously published findings supporting the CLEAR™ Institute’s multimodal approach to non-surgical scoliosis rehabilitation. Case 2 reported 90% compliance with brace wear and home care.

Results

**Figure 3:**
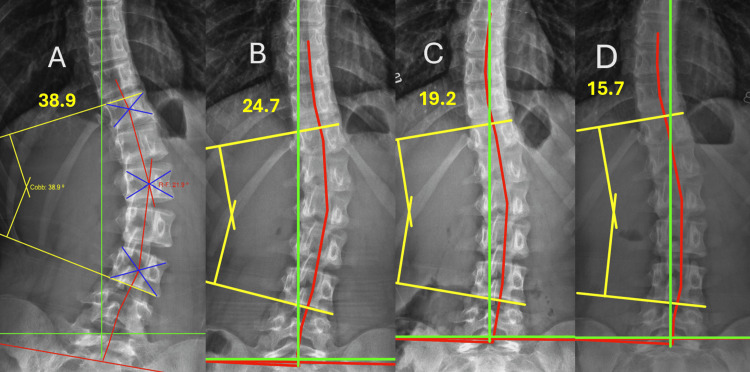
Anterior-posterior (AP) radiographs were obtained at four intervals: pre-treatment (Panel A), prior to intensive care visit 18 on day 10 (Panel B), 6-month follow-up (Panel C), and 12-month follow-up evaluation (Panel D). Yellow lines denote Cobb angle measurements, green lines represent ideal coronal alignment, and red lines illustrate the Risser-Ferguson analysis (Risser 2). These images demonstrate progressive improvement in Cobb angle magnitude and coronal balance over time. All radiographic measurements were performed using PostureRay® software. A PostureRay® study confirms a high degree of inter- and intra-examiner reliability of line-drawing methods, supporting validity. It was also found that there was no statistical difference in average concordance between hand-drawn and PostureRay® measurements in any region of the spine. All radiographs were digitally measured using PostureRay® software, which uses machine-learning-assisted radiographic parameter mensuration [16]. All measurements and radiograph interpretations were performed by the same physician.

Figure 3A depicts the initial radiographs, with a 38.9° Cobb angle. Figure 3B illustrates the results after 18 treatment visits, with a 24.7° Cobb angle and a 37% reduction (these images were taken prior to any treatment provided on the last day of intensive care, with 2 treatments, or 1 full day, remaining). Figure 3C represents the 6-month follow-up after the 2-week intensive program. Figure 3D illustrates the radiographs at the one-year post-treatment evaluation, with a 15.7° Cobb angle, representing a total reduction of 60%.

Post-intensive findings across both cases showed consistent improvements in functional outcomes, chest expansion, angle of trunk rotation, and subjective symptoms assessed using the FRI (Table 1).

## Discussion

The present case series demonstrates that a two-week intensive, non-surgical scoliosis rehabilitation program based on the CLEAR™ Institute protocol can yield meaningful short-term improvements in both sagittal and coronal alignment, consistent with restoration of more physiological spinal biomechanics. These findings reinforce the potential of targeted, frequency-based, multimodal interventions to modify scoliotic deformity. By concentrating repetitive corrective loading into a condensed timeframe, the protocol appears to harness the viscoelastic and adaptive properties of spinal soft tissues, particularly the anterior and posterior longitudinal ligaments, intervertebral discs, and paraspinal musculature [17]. Prior studies have demonstrated that sustained, low-load traction applied for 10-20 minutes can induce measurable ligamentous deformation (creep), improving segmental alignment and facilitating remodelling under controlled mechanical stress [13-18]. The incorporation of neuromuscular re-education techniques, including whole-body vibration, proprioceptive weighting, and dynamic corrective exercise, may enhance the stabilization of these mechanical corrections through improved sensorimotor integration [19,20,21].

This case series achieved radiographic reduction of Cobb angles and subjective improvements in posture and function within two weeks, with sustained gains observed at one-year follow-up. These outcomes support the hypothesis that intensive, high-frequency conservative care can accelerate the timeline of biomechanical adaptation, contrasting with traditional protocols that may require months [10,21]. The rapid response observed here highlights the synergistic benefit of repetitive daily loading and immediate neuromuscular retraining.

Zapata KA et al. (2023) conducted a large U.S. multicentre randomized controlled trial evaluating Schroth-based PSSE in adolescents with mild idiopathic scoliosis [11]. Their findings showed modest improvements in Cobb angle and subject-reported outcomes compared with standard observation. Schroth therapy emphasizes active postural correction, derotation, and respiratory training to improve 3D alignment through neuromuscular re-education. However, the Schroth model is delivered intermittently, typically 2-3 sessions per week over several months, does not incorporate intensive daily frequency or mechanical traction, and may not prescribe concurrent bracing during the active treatment phase. In contrast, the CLEAR™ Institute intensive protocol combines repetitive daily sessions of mirror-image traction, vibration therapy, and proprioceptive re-training over two weeks, accompanied by ScoliBrace® wear for long-term stabilization. This condensed model may amplify the cumulative effects of mechanical loading and neural retraining within a shorter duration, producing more immediate structural corrections while maintaining safety and subject tolerance.

Both approaches share the common goal of restoring optimal spinal alignment and reducing curve progression without surgery. The principal distinction lies in therapeutic density and mechanobiological targeting. Schroth therapy emphasizes neuromotor correction through voluntary activation. The intensive CLEAR™ protocol integrates passive viscoelastic deformation and high-frequency reflexive retraining to achieve comparable or superior radiographic and functional outcomes within a shorter timeframe.

Recent evidence by Nalda A et al. further supports the efficacy of multimodal non-surgical management for moderate-to-severe AIS [10]. Their case series demonstrated clinically significant reductions in thoracic Cobb angle using a combination of CLEAR Institute methods, ScoliBrace®, and scoliosis-specific rehabilitation exercises. Similar to our findings, their program leveraged sustained mechanical traction, targeted postural correction, and proprioceptive training to achieve structural reorganization. However, the Nalda et al. intervention focused on thoracic primary Cobb angles.

This model integrates whole-body vibration and reflexive postural weighting to reinforce sensorimotor pathways responsible for maintaining spinal alignment. Such multimodal reinforcement may accelerate neuromuscular adaptation, allowing corrective postures to be stabilized more efficiently [17].

This case series observed one-year stability in spinal correction, aligning with previous findings that prolonged external bracing, in conjunction with active rehabilitation, helps preserve post-treatment gains [10,21]. This sustained reduction in Cobb angles, particularly in cases of AIS, challenges the historical view that rapid progression is inevitable. These study outcomes suggest that intensive, non-surgical interventions can effectively mitigate scoliotic progression and maintain spinal correction over time. Collectively, these results underscore the potential of short-term intensive protocols to achieve rapid biomechanical and neuromuscular reorganization, offering a clinically efficient, non-invasive alternative for selected AIS subjects.

## Conclusions

This case series demonstrates that, in congruence with previous studies, a two-week intensive non-surgical rehabilitation program based on the CLEAR Institute protocol can produce measurable improvements in coronal alignment in adolescent idiopathic scoliosis (Lenke 5C), with stability at one-year follow-up. The protocol’s high-frequency application of corrective loading and neuromuscular retraining appears to leverage the viscoelastic behavior of spinal ligaments, promoting rapid structural adaptation. Given the limited sample size, future research should include larger longitudinal studies.
